# A novel luminescence-based β-arrestin recruitment assay for unmodified receptors

**DOI:** 10.1016/j.jbc.2021.100503

**Published:** 2021-03-05

**Authors:** Maria Hauge Pedersen, Jennifer Pham, Helena Mancebo, Asuka Inoue, Wesley B. Asher, Jonathan A. Javitch

**Affiliations:** 1 Departments of Psychiatry & Molecular Pharmacology and Therapeutics, Vagelos College of Physicians & Surgeons, Columbia University, New York, New York, USA; 2 Division of Molecular Therapeutics, New York State Psychiatric Institute, New York, New York, USA; 3 NNF Center for Basic Metabolic Research, Section for Metabolic Receptology, Faculty of Health and Medical Sciences, University of Copenhagen, Denmark; 4 Multispan Inc, Hayward, California, USA; 5 Department of Pharmacological Sciences, Tohoku University, Sendai, Japan

**Keywords:** β-arrestin, G protein-coupled receptor (GPCR), bioluminescence, signaling, cell signaling, NanoLuc, signaling pathway, complementation-based assay, Ang II, angiotensin II, AT1R, angiotensin II type 1 receptor, BRET, bioluminescence resonance energy transfer, CHO, Chinese hamster ovary, DAMGO, [D-Ala^2^, *N*-MePhe^4^, Gly-ol]-enkephalin, D2R, dopamine D2 receptor, DOR, δ-opioid receptor, GPCR, G protein-coupled receptor, GRK, G protein-coupled receptor kinase, Hek, human embryonic kidney, HTS, high-throughput screening, KOR, κ-opioid receptor, MOR, μ-opioid receptor, NOP, nociceptin opioid receptor, RLuc8, *Renilla* luciferase 8

## Abstract

G protein-coupled receptors (GPCRs) signal through activation of G proteins and subsequent modulation of downstream effectors. More recently, signaling mediated by β-arrestin has also been implicated in important physiological functions. This has led to great interest in the identification of biased ligands that favor either G protein or β-arrestin-signaling pathways. However, nearly all screening techniques for measuring β-arrestin recruitment have required C-terminal receptor modifications that can in principle alter protein interactions and thus signaling. Here, we have developed a novel luminescence-based assay to measure β-arrestin recruitment to the membrane or early endosomes by unmodified receptors. Our strategy uses NanoLuc, an engineered luciferase from *Oplophorus gracilirostris* (deep-sea shrimp) that is smaller and brighter than other well-established luciferases. Recently, several publications have explored functional NanoLuc split sites for use in complementation assays. We have identified a unique split site within NanoLuc and fused the corresponding N-terminal fragment to either a plasma membrane or early endosome tether and the C-terminal fragment to β-arrestins, which form the basis for the MeNArC and EeNArC assays, respectively. Upon receptor activation, β-arrestin is recruited to the membrane and subsequently internalized in an agonist concentration-dependent manner. This recruitment promotes complementation of the two NanoLuc fragments, thereby reconstituting functional NanoLuc, allowing for quantification of β-arrestin recruitment with a single luminescence signal. Our assay avoids potential artifacts related to C-terminal receptor modification and has promise as a new generic assay for measuring β-arrestin recruitment to diverse GPCR types in heterologous or native cells.

Over the past decade, increased attention has been directed to biased signaling of G protein-coupled receptors (GPCRs) and the possibility of identifying ligands that can selectively target either G protein- or β-arrestin-mediated signaling. As an example, the β-adrenergic receptor ligand carvedilol antagonizes G protein activation but recruits β-arrestin and has been shown to increase survival rates in patients suffering from heart failure (1). At the μ-opioid receptor (MOR), G protein signaling has been proposed to be primarily responsible for analgesia, with side effects such as respiratory depression resulting from β-arrestin-mediated signaling (2, 3), although this paradigm has recently been challenged (4, 5, 6). We have demonstrated that β-arrestin recruitment to the dopamine D2 receptor (D2R) in indirect pathway neurons in the ventral striatum leads to enhanced locomotion, whereas G protein signaling is necessary for incentive behavior (7), further emphasizing the potential importance of biased signaling in more targeted therapeutics.

Agonist induced-GPCR activation leads to GPCR kinase (GRK)-mediated phosphorylation of serine and threonine residues, most notably in the C-terminal tail of receptors but also in the intracellular loops of some receptors, which are the primary receptor recognition regions for β-arrestin binding. In addition to its role in signaling, β-arrestin also regulates receptor internalization and for some receptors stays bound to endosomes where receptor-mediated signaling may continue to occur (8).

The most well-established techniques to investigate β-arrestin recruitment are the PathHunter, Tango, LinkLight, and bioluminescence resonance energy transfer (BRET) assays. The PathHunter assay is an enzyme complementation assay in which split enzyme fragments are fused to the receptor and to β-arrestin and complementation of the functional enzyme creates a chemiluminescence readout (9). The Tango-GPCR assay is a reporter gene assay where a transcription factor fused to the receptor C terminus is cleaved off by a protease-tagged β-arrestin, leading to the expression of a reporter that creates a luminescence readout (10, 11). LinkLight uses a modified luciferase attached to β-arrestin that is cleaved off by a protease fused to the C terminus of the GPCR of interest, thereby activating the luciferase and producing light (12). BRET between a receptor fused at its C terminus to a luciferase donor and β-arrestin tagged with fluorescent protein acceptor is also commonly used to measure β-arrestin recruitment (13). Notably, all of these β-arrestin assays require that fusion tags be directly attached to the C terminus of receptors of interest, which could alter GRK, arrestin, or other protein interactions with the receptors and therefore impact signaling. The only method that does not rely on modified receptors is the microscopy-based Transfluor assay where β-arrestin fused to a fluorescent protein is visualized at the membrane (14). However, with this technique, it is difficult to quantify ligand efficacy and potency or to scale for screening ligands.

In contrast, we previously introduced a modified BRET assay in which a *Renilla* luciferase 8 (RLuc8) donor is anchored to the membrane instead of attached to the receptor (13). In this assay, which is based on “bystander” BRET, β-arrestin fused to a venus acceptor is translocated from the cytosol to the unmodified receptor at the plasma membrane, leading to an increase in BRET by proximity between the membrane-anchored donor and acceptor attached to β-arrestin. This assay incorporated yeast-derived “helper” peptides that are attached to the donor and acceptor, thereby enhancing the affinity of the interaction between β-arrestin and the plasma-membrane anchor (15). This bystander BRET format was later adapted to a BRET2 format (16). BRET assays require a specialized dual output microplate reader and filter sets compatible with BRET. The previously used BRET assays also (13) use RLuc8 instead of the more recently reported novel luciferase NanoLuc developed by Promega, which has the advantages of being smaller and significantly brighter compared with other known luciferases (17).

Recently, a split NanoLuc, known as NanoBiT, which is commercially available from Promega (Fig. S1A), has been used successfully in a direct β-arrestin recruitment assay in which a small peptide fragment, referred to as SmBiT, is attached to the receptor of interest and the larger fragment, referred to as LgBiT, is attached to β-arrestin (18). A slightly modified version of NanoBiT has proven to be effective for compound library screening as seen in a direct recruitment assay between D2R and Gα_i1/o_ (19). Thus, we expected that the NanoBiT system would readily be adapted to our bystander assay (13) by fusing SmBiT to our membrane anchor instead of directly to the receptor and LgBiT to β-arrestin, thereby substituting BRET in our assay with complemented NanoLuc detection. However, to our surprise, the NanoBiT adaptation of our assay failed to detect β-arrestin recruitment to the membrane upon receptor activation.

Here, we report a novel NanoLuc split that, unlike NanoBiT, is suitable for the desired NanoLuc-complementation-based β-arrestin membrane recruitment assay where the membrane anchor is attached to the N-terminal NanoLuc fragment (referred to as MeN) and β-arrestin is attached to the C-terminal NanoLuc fragment (referred to as ArC). Our assay, which we refer to as MeNArC, does not rely on receptor modification or helper peptides and provides a simple luminescence output reading of β-arrestin recruitment as an alternative to the bystander BRET assay to investigate receptors in a more native format. We also adapted the assay to measure β-arrestin recruitment to early endosomes, referred to as EeNArC, demonstrating that the assay can detect events in specific cell compartments other than the plasma membrane. We anticipate that these novel assays will serve as general approaches to screen compounds for β-arrestin recruitment to unmodified GPCRs *in vitro* and potentially *in vivo*.

## Results

### Validation of a unique NanoLuc split for complementation assays

**Figure 1:**
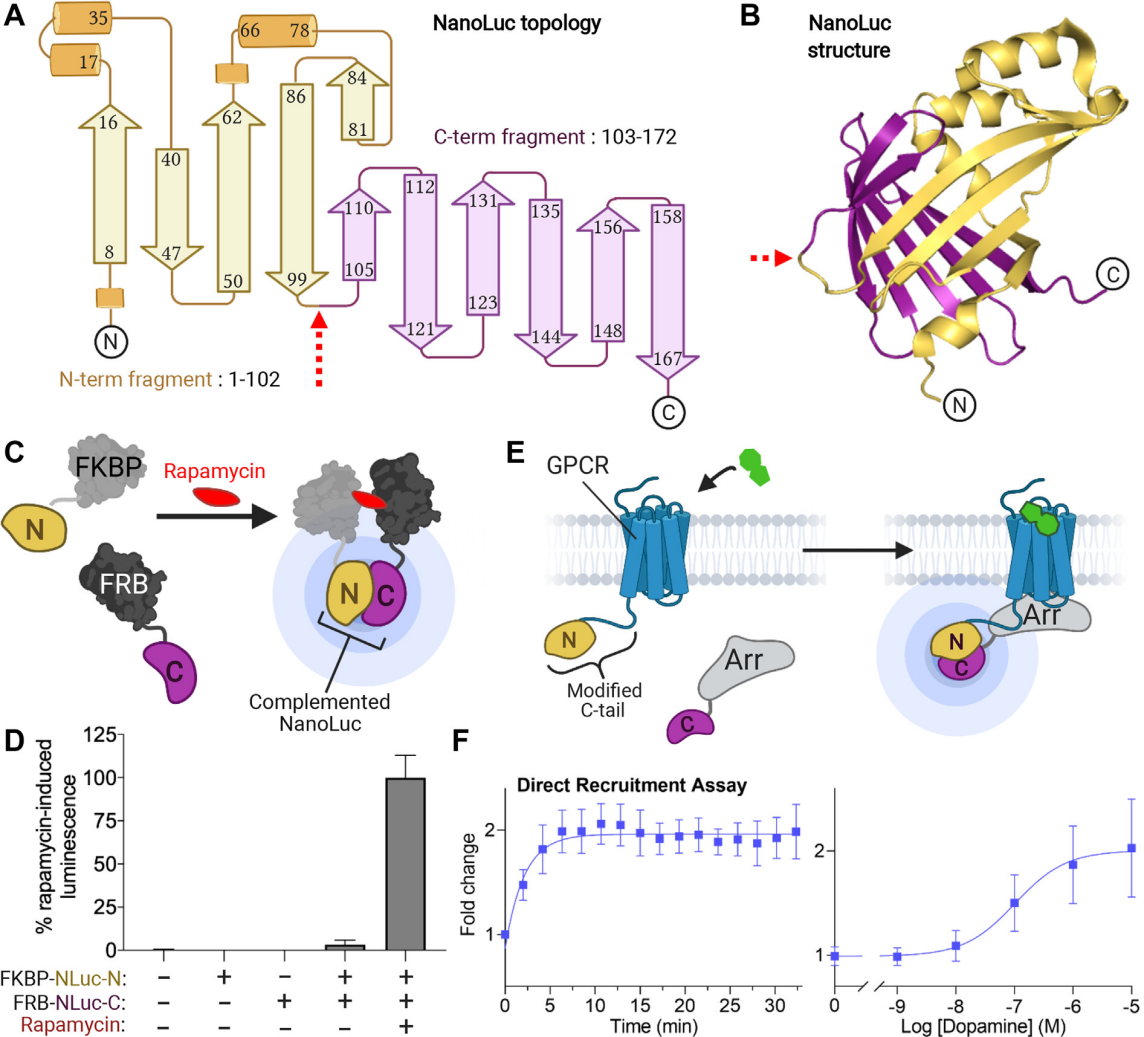
**Validation of a novel NanoLuc split for use in complementation-based assays.***A,* topology of NanoLuc with the unique split site shown with a *red arrow* and the corresponding N-terminal (residues 1–102) and C-terminal (residues 103–172) split fragments shown in *yellow* and *purple*, respectively. The topology diagram is derived from that generated by pro-origami (34). *B*, structure of NanoLuc (PDB #5IBO) rendered in PyMol (35) showing the N- and C-terminal components in *yellow* and *purple*, respectively, and the split site depicted by a *red arrow*. *C*, schematic overview of rapamycin-induced dimerization of FKBP and FRB fused to the N- and C-terminal NanoLuc fragments, respectively. *D*, quantification of luminescence from Hek293 cells in the presence of coelenterazine H for the following conditions: cells with no transfection, cells transiently transfected with constructs coding for either FKBP fused to the N-terminal NanoLuc fragment or FRB fused to the C-terminal NanoLuc fragment alone, and cells transfected with both constructs with and without 50 nM rapamycin treatment. *E*, schematic describing the direct recruitment assay where the N-terminal and C-terminal NanoLuc fragments are fused to the C terminus of a receptor and to the N terminus of β-arrestin, respectively. *F*, direct recruitment assay with D2R. Time course after dopamine addition on the left yielded a fold increase in luminescence of 1.96 ± 0.02 that plateaued ∼10 min after 10 μM dopamine treatment and dose–response curve on the right (pEC50: −6.99 ± 0.24). All data represent mean ± SD of three to five independent experiments with triplicate determinations.

As described above, we initially used the NanoBiT system (Fig. S1*A*) in our previously developed assay (13) to detect membrane recruitment of β-arrestin. However, the beta 2 adrenergic receptor (β2AR), D2R, and AT1R all failed to produce any increase in luminescence in this assay upon agonist stimulation (Fig. S1*B*). We reasoned that the nature of the NanoBiT split (Fig. S1*A*) might prevent proper complementation when used in our bystander membrane recruitment assay and therefore designed a new split site within NanoLuc using the crystal structure of the protein as a guide (20). We selected a site in a loop region that divides NanoLuc into two almost equally sized fragments (N-terminal fragment, amino acids 1–102; C-terminal fragment, amino acids 103–172), without disrupting any secondary structural element, in an effort to select fragments that would fold independently and that could efficiently compliment when brought together at the membrane (Fig. 1, *A* and *B*). This site was similar but not identical to another NanoLuc split site used previously in a direct-recruitment assay (21).

To verify that our split fragments can form a fully functional protein capable of luminescence when complemented, we first attached the N- and C-terminal NanoLuc fragments to FKBP (FK506- and rapamycin-binding protein) and FRB (FKBP-rapamycin-binding domain) respectively, which can readily dimerize with high affinity upon addition of rapamycin (Fig. 1*C*) (22). When transfected alone, the fragments did not yield any luminescence over the baseline observed with mock transfected cells (Fig. 1*D*). Cotransfection of both fragments yielded a small basal luminescence signal that was increased ∼30-fold by the addition of rapamycin (Fig. 1*D*), suggesting only minimal spontaneous assembly of the fragments but efficient complementation when brought into proximity, demonstrating that the new fragments are suitable as a tool for complementation assays. We further validated their use for complementation-based GPCR assays in a direct recruitment assay with β-arrestin, as we have done previously with the D2R receptor in a BRET setup (13), in which the N-terminal Nanoluc fragment was attached to the C-terminal tail of D2R and the C-terminal fragment was fused to the N terminus of β-arrestin2 (Fig. 1*E*). In this configuration, the endogenous agonist dopamine produced a time- and dose-dependent increase in luminescence (Fig. 1*F*).

### Detection of β-arrestin plasma membrane recruitment by class A receptors

**Figure 2:**
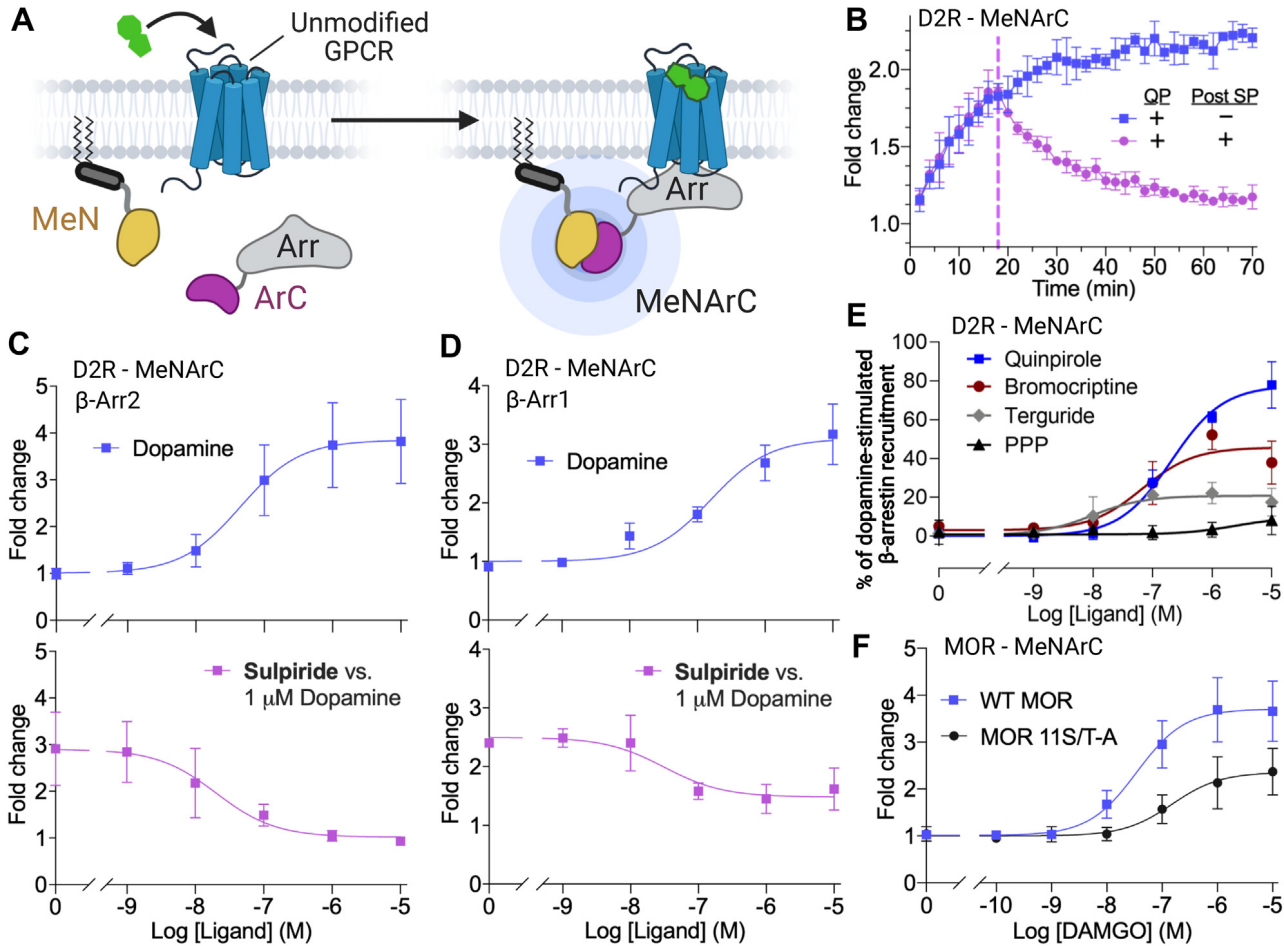
**MeNArC assay with unmodified class A receptors.***A,* schematic overview of the arrestin membrane-recruitment assay. The N-terminal NanoLuc fragment is fused to a doubly palmitoylated fragment of GAP43, thereby tethering it to the plasma membrane (MeN), and the C-terminal NanoLuc fragment is fused to the N terminus of β-arrestin (ArC). *B*, β-arrestin2 recruitment shown in fold change of luminescence for D2R with time course for 1 μM quinpirole-induced β-arrestin2 recruitment in the absence (*blue*) or presence (*purple*) of 10 μM sulpiride injected 18 min after agonist treatment. Steady state is reached after ∼40 min. Dose–response curves of (*C*) β-arrestin2 and (*D*) β-arrestin1 recruitment by D2R with dopamine alone (*blue line*, top plots) and with 1 μM dopamine post treatment with increasing concentration of the antagonist sulpiride (*purple line*, bottom plots). Experiments were carried out with D2R cotransfected with GRK2. The pEC50 for β-arrestin2 with quinpirole was: −6.8 ± 0.16 and IC50 sulpiride: −7.50 ± 0.35. For β-arrestin1 the pEC50 for quinpirole: −7.35 ± 0.17 and IC50 sulpiride: −8.37 ± 0.95. *E*, β-arrestin2 recruitment tested with the D2R partial agonists quinpirole, bromocriptine, terguride, and PPP yielding pEC50 ± SD compared with dopamine of −6.68 ± 0.09, −7.18 ± 0.32, −8.02 ± 0.25, and −5.61 ± 1.01, respectively. *F*, dose–response curves of β-arrestin2 recruitment by wild-type (WT) MOR (*blue*, *square*) and MOR 11S/T-A (*black*, *circle*) with increasing concentration of agonist DAMGO; pEC50 ± SD: WT MOR, −7.46 ± 0.11, and MOR 11S/T-A, −6.82 ± 0.19. Global fits from three to eight independent experiments each performed in triplicate. Error bars represent SD.

We next incorporated the validated NanoLuc split components into our β-arrestin recruitment assay with unmodified receptors where the N-terminal fragment of NanoLuc is anchored to the plasma membrane through a double palmitoylated domain of GAP43 (MeN) and where the C-terminal fragment is fused to the N terminus of β-arrestin1 or β-arrestin2 (ArC) (Fig. 2*A*). The helper peptides that were used in our original BRET assay were not used in the current split Nanoluc configuration (13). To test our MeNArC assay, we used D2R and MOR, which are prototypical class A receptors based on their agonist-induced β-arrestin-binding profile after internalization (23), as β-arrestin readily dissociates from these receptors upon receptor endocytosis. When D2R was used in this assay, in contrast to the results when the NanoBiT fragments were used (Fig. S1*B*), we observed a robust increase in luminescence upon agonist treatment that plateaued after ∼40 min (Fig. 2*B*), consistent with β-arrestin recruitment to the plasma membrane. Addition of the D2R antagonist sulpiride 18 min after receptor stimulation with the agonist quinpirole led to a strong decay in luminescence down to baseline (Fig. 2*B*), indicative of nearly complete reversal of complementation between the NanoLuc fragments upon receptor inhibition, suggesting that the affinity between the fragments is relatively low. Both β-arrestin isoforms in the assay gave similar fold increases of 3.1 ± 0.14 and 3.4 ± 0.20 for β-arrestin1 and 2, respectively (Fig. 2, *C* and *D*), with similar potencies (β-arrestin1 EC50 156 nM ± 0.16; β-arrestin2 EC50 74 nM ± 0.18). While the MeNArC assay also can be used with the splits in the reverse orientation, we observed a lower fold increase in luminescence upon receptor activation (Fig. S2), and therefore we used the assay in the preferred orientation described above for all subsequent experiments. For further study of the assay, we proceeded with β-arrestin2, which is the β-arrestin isoform most typically used in β-arrestin assay development.

To assess the MeNArC assay’s dynamic range, we tested D2R with the high-efficacy partial agonist quinpirole, medium-efficacy partial agonists bromocriptine and terguride, and the low-efficacy partial agonist (3-hydroxyphenyl)-N-propylpiperidine (PPP), giving an efficacy compared with dopamine of 78%, 46%, 24%, and 10%, respectively, similar to previously published data using the BRET direct recruitment assay (24). These results show that the MeNArC assay is also capable of measuring different degrees of partial agonism of unmodified receptors (Fig. 2*E*).

To further validate the MeNArC assay, we also tested MOR compared with a previously reported MOR mutant (MOR 11S/T-A) with impaired β-arrestin binding (25). In MOR 11S/T-A, 11 serine and threonine residues in the C-terminal tail of the receptor are replaced with alanine, preventing C-terminal phosphorylation and therefore inhibiting recruitment of β-arrestin. The fold change of MOR compared with MOR 11S/T-A in response to activation by DAMGO ([D-Ala^2^, *N*-MePhe^4^, Gly-ol]-enkephalin) was 3.7 ± 0.10 and 2.4 ± 0.11, respectively (Fig. 2*F*), decreasing the efficacy to 65% for MOR 11S/T-A. Previously, MOR 11S/T-A had been tested in a direct recruitment assay giving an efficacy of 42% compared with WT. In both assays the MOR 11S/T-A mutant exhibits decreased efficacy, demonstrating that our complementation assay can differentiate between receptors with known differences in β-arrestin recruitment and that the assay is sensitive to the receptor’s affinity for β-arrestin. These experiments verify the system’s direct link between β-arrestin recruitment and the luminescence signal.

### Detection of β-arrestin plasma membrane and early endosomal recruitment by a class B receptor

**Figure 3:**
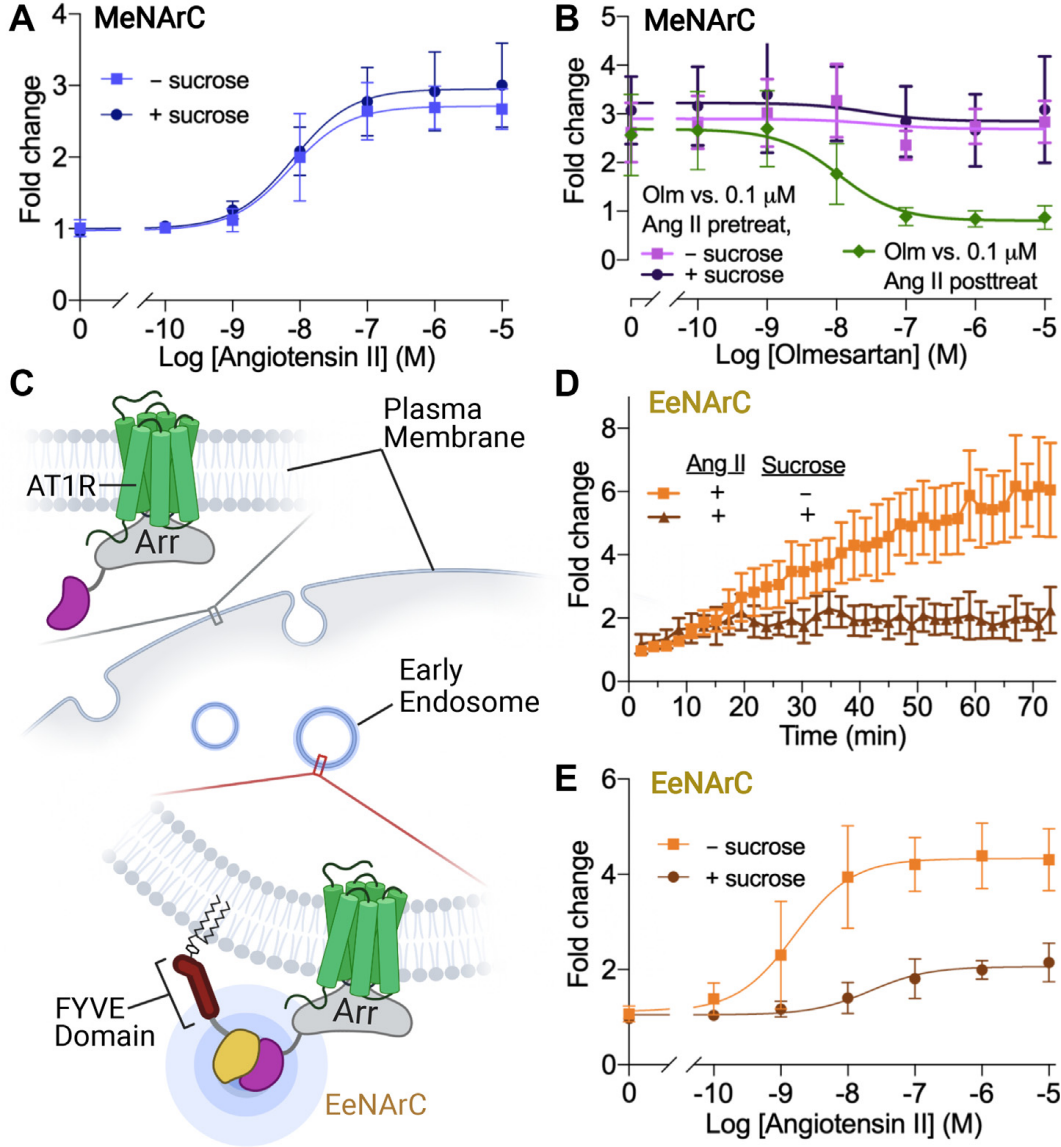
**MeNArC and EeNArC assay with an unmodified class B receptor.***A,* β-arrestin recruitment to the membrane by AT1R stimulated with the endogenous agonist Ang II shown in fold change in the absence (*light blue squares*; pEC50 ± SD: −8.1 ± 0.12) or presence of 450 mM sucrose added 20 min pre-agonist treatment to inhibit internalization (*dark blue circles*; pEC50 ± SD: −8.14 ± 0.19). *B*, AT1R β-arrestin recruitment with the antagonist olmesartan treated with 0.1 μM Ang II post- (*green*) and pretreatment (*light purple*) as well as 450 mM sucrose and agonist pretreatment (*dark purple*). *C*, schematic overview of the EeNArC assay where β-arrestin recruitment is measured specifically at early endosomes. Here, the N-terminal NanoLuc fragment is fused to the FYVE domain of endofin, thereby tethering it to the early endosome (EeN), and the C-terminal NanoLuc fragment is fused to the N terminus of β-arrestin (ArC). *D* and *E,* EeNArc assay with AT1R shown in fold change of luminescence with (*D*) time course for 10 μM Ang II-induced arrestin recruitment in the absence (*orange*) or presence (*brown*) of 450 mM sucrose and (*E*) dose−response curves for Ang II alone (pEC50 ± SD: −8.79 ± 0.22) or pretreated with 450 mM sucrose (pEC50 ± SD: −7.66 ± 0.30). Global fits from three to four independent experiments each performed in triplicates. Error bars represent SD.

To investigate the versatility of the MeNArC assay, we further tested its use with the class B angiotensin II type 1 receptor (AT1R). Unlike class A receptors, AT1R and other class B receptors bind β-arrestin with higher affinity and form longer-lived complexes that persist after endocytosis (23). AT1R led to a robust increase in luminescence upon stimulation with the agonist Angiotensin II (Ang II) with a fold change of 3.0 ± 0.12 (Fig. 3*A*). The addition of sucrose, a known inhibitor of receptor endocytosis (26), had no effect on agonist response, suggesting that the observed signal is generated at the plasma membrane (Fig. 3*B*).

To investigate the reversibility of β-arrestin recruitment to a class B receptor, we tested AT1R with the antagonist olmesartan. Interestingly, agonist-induced β-arrestin recruitment by AT1R could not be reversed upon subsequent addition of increasing concentrations of olmesartan, either in the absence or presence of sucrose (Fig. 3*B*). In contrast, the class A receptor D2R was inhibited by antagonist in the same assay (Fig. 2*B*), showing the reversibility of activation. Notably, we were able to prevent the agonist-induced response at AT1R with olmesartan treatment when the antagonist was added before agonist (Fig. 3*B*). To understand whether this was due to rapid internalization of AT1R-β-arrestin complexes that might become inaccessible to inhibition by olmesartan, we inhibited internalization with sucrose, but this did not enhance olmesartan’s ability to inhibit the agonist-induced response (Fig. 3*B*). We therefore reasoned that this irreversibility reflects the much higher affinity of β-arrestin interaction with AT1R once the receptor C tail is phosphorylated and engaged with β-arrestin (23). Like what is reported for the beta 2 adrenergic receptor-V2R chimera, we infer that even after blocking core engagement of AT1R with β-arrestin by addition of the antagonist, the “hanging” interaction with the phosphorylated receptor C tail is sufficient to maintain the interaction (27), in contrast to MOR and D2R where core engagement and therefore continued agonist binding are essential for β-arrestin interaction.

Because class B receptors form long-lived complexes with β-arrestin that can be imaged after internalization (23), we sought to adapt our NanoLuc complementation assay to also measure β-arrestin recruitment to early endosomes containing internalized AT1R. For this assay, referred to as EeNArC, the ArC component described above is coexpressed with the N-terminal NanoLuc fragment attached to the FYVE domain of endofin (EeN), which was shown previously to selectively bind endosomes and used in a direct receptor recruitment assay (16) (Fig. 3*C*). Stimulation of the receptors with Ang II resulted in a robust increase in luminescence over time that reached a plateau after 60 min, with an EC50 of 1.6 nM ± 0.22, consistent with β-arrestin recruitment to early endosomes (Fig. 3*D*). As expected, the agonist effect was dramatically blunted by sucrose for the EeNArC assay (Fig. 3, *D* and *E*), but not for the MeNArC assay (Fig. 3*B*), demonstrating that the former is specific to events that occur at early endosomes and thus dependent on internalization (Fig. 3, *D* and *E*). The MeNArC and EeNArC assays were also performed in human embryonic kidney (Hek)293 cells without cotransfection of any receptor, and no response was observed for dopamine, Ang II, or DAMGO, establishing the specificity of the assay for the transfected GPCRs (Fig. S3).

### Development of a polycistronic MeNArC expression vector for detecting β-arrestin recruitment in stably transfected CHO-K1 cells for use in high-throughput screening

**Figure 4:**
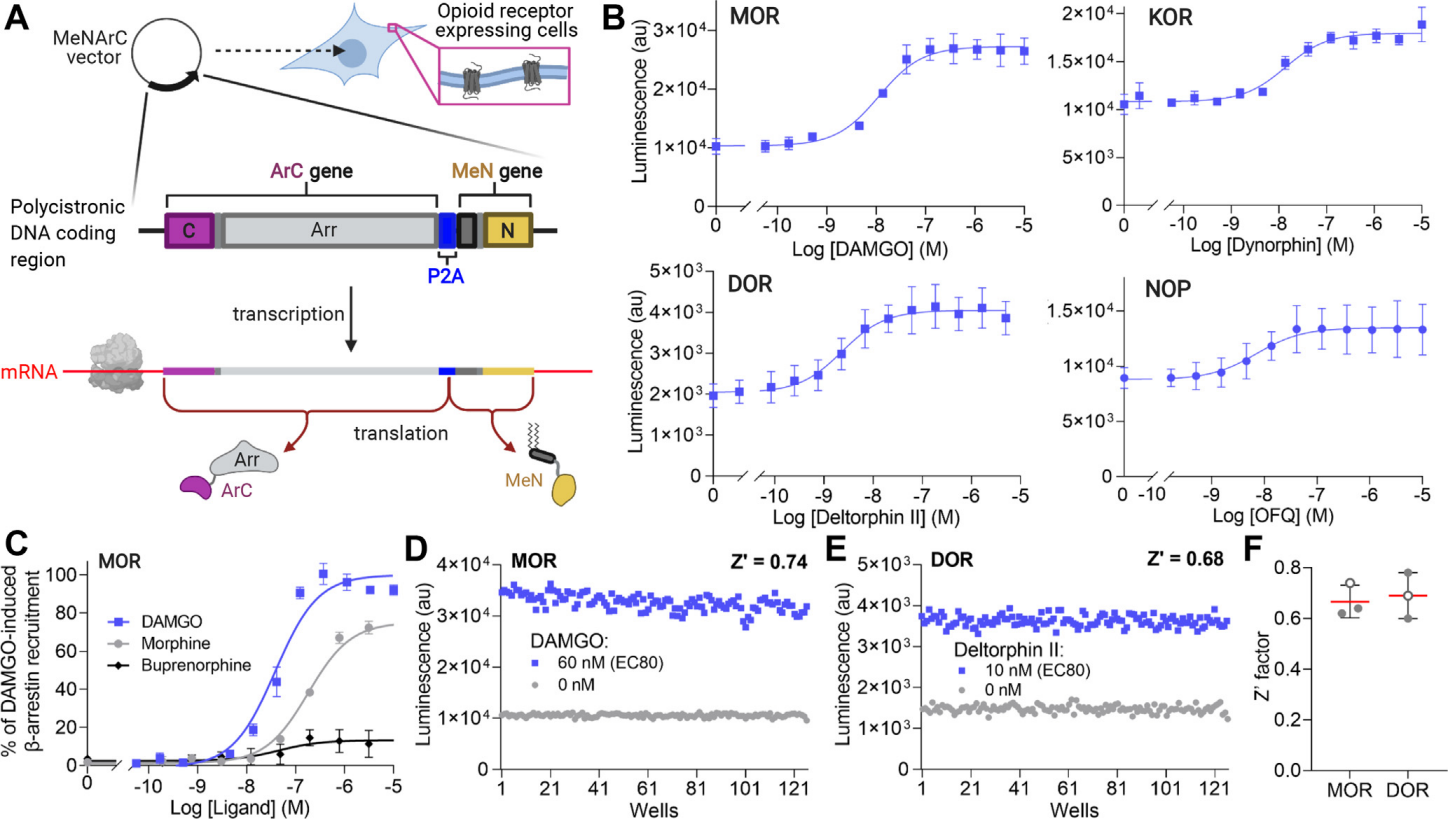
**Generation of stable cell lines using a MeNArC polycistronic vector for use in high-throughput screening.***A,* schematic representation of the polycistronic MeNArC vector containing the coding region for the ArC and MeN components separated by a P2A ribosomal slippage sequence that allows for the expression of both of the proteins from a single vector. The vector was stably integrated into CHO cells expressing each of the opioid receptors to carry out the MeNArC assay. *B*, dose–response curves of the MeNArC assay for the MOR, KOR, DOR, and NOP cells showing increase in luminescence (au) with increasing concentration of agonist; KOR (pEC50: −7.86 ± 0.10), MOR (pEC50: −7.7 ± 0.15), DOR (pEC50: −8.63 ± 0.12), or NOP (pEC50: −8.15 ± 0.24), stably transfected into CHO-K1 cells together with the β-arrestin2 version of the polycistronic ArC-P2A-MeN plasmid. *C*, β-arrestin2 recruitment tested with the MOR partial agonists morphine and buprenorphine normalized to the full agonist DAMGO. *D* and *E*, representative Z’-factor data from one data set conducted with (*D*) MOR and (*E*) DOR on a 384-well plate in the absence (*gray*) or presence of agonist (*blue*). *F*, comparison of Z’-factor data compiled from three individual experiments showing mean values (*red middle line*) of 0.67 ± 0.06 and 0.69 ± 0.09 for MOR and KOR, respectively, and SD. The open circles represent the values determined from the plots shown in (*D* and *E*).

For the experiments described above, each component of the MeNArC assay, as well as the receptor, was expressed transiently from individual expression vectors. While this approach is suitable for such *in vitro* experiments, it is challenging to use several vectors for generating cell lines stably expressing these components or for potential use *in vivo*. Thus, we designed a single polycistronic expression vector encoding both the ArC and MeN components separated by the self-cleaving porcine *Teschovirus*-1 2A (P2A) peptide sequence (Fig. 4*A*) (28). When introduced into cells expressing receptor, the P2A sequence induces ribosomal skipping during translation, thereby effectively cleaving the P2A peptide and generating both the ArC and MeN proteins from a single expression construct.

To show that our polycistronic MeNArC vector could be used to detect β-arrestin recruitment in receptor expressing cells, we stably integrated the vector into Chinese hamster ovary (CHO)-K1 cell lines stably expressing each of the wild-type opioid receptors without any C-terminal modifications (see Experimental procedures). The μ, δ, κ, and nociceptin opioid receptor (MOR, DOR, KOR, and NOP, respectively) expressing cells all exhibited robust dose–response curves with their respective full agonists (Fig. 4*B*). We also showed that the MOR cell line treated with the partial agonists morphine and buprenorphine gave pEC50 values of −6.74 ± 0.18 and −7.36 ± 0.98, with efficacy levels compared with full agonist DAMGO of ∼60% and ∼10% respectively, consistent with the results of a previously published direct recruitment assay (Fig. 4*C*) (29).

To determine whether the agonist-induced response of the MeNArC assay is sufficiently robust for high-throughput screening (HTS), we determined the Z’-factor for both the MOR and DOR stable cell lines in 384-well plates in the absence or presence of EC80 concentrations of agonist (Fig. 4, *D* and *E*). From three independent experiments, the average (± standard deviation) Z’-factor for MOR and DOR was 0.67 ± 0.06 and 0.69 ± 0.09, respectively, which are well within the acceptable Z’-factor range of 0.5–1, showing that our assay is highly suited for HTS (30).

## Discussion

We have developed a novel NanoLuc complementation assay that measures β-arrestin recruitment to the plasma membrane and early endosomes with nonmodified receptors using a simple luminescence output. The most widely used β-arrestin assays available all rely on receptors with C-terminal modifications, which can have unwanted effects on the outcome, as the C-terminal tail of the receptor is also the primary recognition site for β-arrestins in most receptors, which might alter receptor–arrestin interactions or otherwise impact receptor interactions with other proteins (31). As noted above, the only other available assays that use unmodified receptors are the Transfluor assay, where the reading output is microscopy-based, and the aforementioned BRET method, both of which are associated with the limitations described in the introduction.

Complementation-based luciferase split assays have been widely used in the last decade to detect protein–protein interaction in multiple biological systems; NanoLuc has advantages in both decreased size and increased brightness (17). Several NanoBiT-based GPCR complementation methods have been developed, including the detection of direct β-arrestin recruitment, direct G protein interaction, and receptor internalization, all of which have SmBiT fused to the C terminus of the receptor (18, 19, 32). Curiously, we found that the NanoBiT system, when adapted to the β-arrestin membrane recruitment assay, failed to show a response with any of the three receptors tested, including D2R (Fig. S1*B*). Why these split fragments work with the direct recruitment assay but not the membrane recruitment assay is unclear, but we speculate that this might result from unfavorable orientation of the attachments of the large and small fragments. Thus, we split NanoLuc into two roughly equal sized fragments, which were successfully implemented into our assay. We tested our membrane recruitment assay with the opioid receptors, D2R and AT1R. The MeNArC assay was reversible for class A receptors, which suggests it could be used to investigate inverse agonism in receptors with sufficient constitutive activity. We further adapted the method to detect β-arrestin recruitment to early endosomes, which was dependent on receptor internalization, providing an opportunity to study β-arrestin trafficking upon receptor activation. We also generated a single plasmid containing both MeNArC components for the creation of stably transfected cell lines that can be used for HTS. However, it is important to note that small molecule libraries can contain fluorogenic compounds that can interfere with luminescence and show up as false inverse agonists, highlighting the importance of using parallel approaches for compound screening.

Although our recruitment assays employ unmodified receptors, we cannot rule out action of a tested agonist on endogenous receptors or possible off-target receptors when tested in native tissue unless knockout models are available. Indeed, in future studies it will be interesting to test the utility of MeNArC and EeNArC in primary cell cultures for measuring β-arrestin recruitment to endogenous receptors. Whether this is successful will likely depend on the level of endogenous receptor expression. Finally, we note that while our assay reports on β-arrestin recruitment, like all β-arrestin recruitment assays, it does not measure β-arrestin “activity” and therefore does not directly report on downstream signaling, which can involve several different pathways such as ERK and Src (33).

In conclusion, we have developed β-arrestin membrane and endosomal recruitment assays using complementation of novel NanoLuc fragments with the potential of serving as a generic and readily adapted screening method for detecting β-arrestin recruitment to unmodified receptors.

## Experimental procedures

### Compounds

Dopamine hydrochloride (#H-8502), S-(-)3-PPP (#P-103), Terguride (#T-134), angiotensin II (#A9525), and buprenorphine hydrochloride (#B9275) were purchased from Sigma-Aldrich; sulpiride (#0895), quinpirole (#1061), bromocriptine (#0427), olmesartan (#4616), Dynorphin B (#3196), naloxone hydrochloride (#0599), WN552122 (#1038), and rimonabant hydrochloride (#0923) from Tocris; DAMGO (#024-10) from Phoenix Pharmaceuticals, and rapamycin (#tlrl-rap) from Invivogen and morphine sulfate (#M1167) from Spectrum Chemicals.

### Plasmid constructs

The FRB, FKBP, and MeNArC sensor plasmids were constructed using standard techniques in molecular biology. Briefly, primers were designed for PCR production with 15–20 overlapping base pairs between the constructs that were to be fused together in further PCR production until the desired constructs were made. For end primers either restriction sites were introduced, which allowed for digestion of the purified PCR products and further ligation into pcDNA3.1+ (Invitrogen) or the generic end primers CMV-F and BgH-R were used where restriction sites already where present that could be used. Construction of the early endosome markers was carried out using Gibson technology (NEB).

NanoLuc was split to create an N-terminal part (N1) with amino acids 1–102 and a C-terminal part (N2) with amino acids 103–172. N1 and N2 were attached *via* an eight amino acid flexible linker at the C-terminal end of FKBP and N2 at the C-terminal end of FRB (FKBP-rapamycin-binding domain of mTOR), both with a signal peptide (MKTIIALSYIFCLVFA) and myc (EQKLISEEDL) tag at the N-terminal of FRB/FKBP. NanoLuc was codon optimized for mammalian expression (see Fig. S4 for sequence). All receptor constructs were in pcDNA3.1+. Human D2R had the signal peptide and a FLAG (ADYKDDDDA) tag attached to the N-terminus used previously (13). Mammalian AT1R, MOR WT, and MOR 11S/T-A had a signal peptide and snap tag attached to the N-terminus. The constructs for the direct and indirect β-arrestin recruitment assays were made by exchanging the donor and acceptor with the NanoLuc splits from the BRET β-arrestin method previously described (13) as well as removal of the helper peptides SH3 and Sp1 to make D2R-linker-N1, mem-linker-N1 (membrane tethered N-terminal split, MeN), and N2-linker-β-arrestin2 (β-arrestin tethered C-terminal split, ArC). The polycistronic vector was made by inserting the 22 amino acid P2A cleavage peptide between the sequence of MeN and ArC, creating ArC-P2A-MeN in pcDNA3.1+. The plasmid coding for GRK2 was obtained from David Sibley. For the CHO-K1 stable cell lines, the human MOR, KOR, DOR, and NOP were all inserted into the pMEX2 vector from Multispan (puromycin resistant). All products used for molecular biology were purchased from NEB. The full list of primers, products, volumes, PCR cycles, and construct sequences used for molecular biology can be found in Tables S1–S3, respectively.

To compare Promega’s NanoBiT system, β-arrestin2 was inserted at the C-terminal end of LgBiT using Promegas pBiT1.1-N vector (Promega Cat. #N2014) and the doubly palmitoylated fragment of GAP43 was inserted at the N-terminal of SmBiT using the pBiT2.1-C vector.

### Cell culture and transfection

Hek293 cells (ATTC CRL-1573) were grown in DMEM + GlutaMAX-I (Gibco, Invitrogen) with 10% fetal bovine serum (Cornig #35-010) and 1% penicillin/streptomycin (Cornig #30-002) at 37 °C with 5% CO_2_. Cells were transfected with either lipofectamine 3000 2 μl/1 μg DNA (Sigma) or polyethylenimine (PEI; linear, MW 25,000; Polysciences, cat. No. 23966-2) at a 2:1 ratio (PEI:DNA) in growth medium. The DNA concentrations for transfection in a 10 cm^2^ plate seeded with 3 × 10^6^ cells for initial split nanoluc testing were 1.5 μg of both FKBP-Nanoluc N-term and FRB-Nanoluc C-term; for β-arrestin recruitment, 1.5 μg receptor, 1.5 μg MeN/EeN, 1.5 μg ArC ± 4.8 μg GRK2, and empty vector DNA was added to reach a final DNA concentration of 15 μg.

CHO-K1 cell lines stably expressing the opioid receptors were further transfected with ArC-P2A-MeN and single clones were chosen with an intermediate luminescence level. The cell lines were grown in DMEM/HAM F12 with 1% L-glutamine (Hyclone SH30023.01), 10% fetal bovine serum, 10 μg/ml puromycin (invivogen ant-pr-5), 800 μg/ml G418 (Corning 30-234-CI), and 1% penicillin/streptomycin at 37 °C with 5% CO_2_.

### β-Arrestin complementation assay

For the initial testing of the NanoLuc split site, 50 nM rapamycin was added to the culture medium 1 day after transfection. Two days after transfection, the Hek293 cells were washed with Dulbecco’s phosphate-buffered saline (D-PBS) and resuspended in 7.5 ml D-PBS containing 5 mM glucose. In total, 50 μl/well was seeded into a 96-well black-white iso plate (PerkinElmer) to reach a cell count of 25–40K cells/well and supplemented with 5 μM of Furimazine (Promega #N1120) or coelenterazine-H (Dalton #50909-86-9). After 5 min incubation at room temperature with a foil cover, ligand was added, and luminescence was measured every 2 min for kinetics data or after 40 min for dose–response curves using a PHERAstar FS (BMG labtech) plate reader.

The stably transfected CHO-k1 cells were seeded out 8K cells/well into a white opaque bottom Poly-D-lysine-coated 384 well plate (Corning BioCoat #356661). Cells were washed with HBSS and incubated with 5 μM coelenterazine-H for 5 min before compound addition. Luminescence was read 20 min after incubation on a Flexstation III.

### Data analysis

Data were analyzed using GraphPad Prism Version 8. Transient transfection data was normalized to fold change of basal luminescence and stable cell line data were directly pooled raw luminescence data. For partial agonism, partial agonist efficacy was normalized to the full agonist of the given receptor. All data is shown with mean ± SD values. Figures were prepared using Biorender.

## Data availability

The majority of data that support the findings of this study are included within the article. Data not displayed in the article are available from the corresponding author upon reasonable request: Jonathan A. Javitch, javitch@nyspi.columbia.edu, Departments of Psychiatry & Molecular Pharmacology and Therapeutics, Vagelos College of Physicians & Surgeons, Columbia University, New York, NY and Division of Molecular Therapeutics, New York State Psychiatric Institute, New York, NY.

## Supporting information

This article contains supporting information.

## Conflict of interest

This technology has been licensed to Multispan by Columbia University and is commercially available under the MultiScreen brand name. The authors Jennifer Pham and Helena Mancebo are employed by Multispan.
