# Supplementary material for: A novel luminescence-based β-arrestin recruitment assay for unmodified receptors
Source: J Biol Chem. 2021 Mar 5;296:100503. doi: 10.1016/j.jbc.2021.100503 (PMC8027564; doi:10.1016/j.jbc.2021.100503)
Supplement: Tables S1 to S3 [file mmc1.pdf]

Supplementary information

Table 1 - primers			
**Construct #**	**construct**	**Primer name**	**Primer sequence**
1	FKBP_N1	KpnI_SP_myc fwd	5'-GCTTGGTACCATGAAGACGATCATCGCCCTGAGCTACATCTTCTGCCTGGTGTTCGCCGAGCAGAAGCTGATCTCCGAAGAGGACCTGGATCCAGTGCAGGTGGAGACCATCTC-3'
1		FKBP_link_N1 Rev	5'-CTTCGAGTGTGAAGACCATCGCCCCCGCCGCCCCCGCCTCGAGCTTTTCCAGTTTTAGAAGC-3'
1		FKBP_link_N1 fwd	5'-GCTTCTAAAACTGGAAAAGCTCGAGGCGGGGGCGGCGGGGGCGATGGTCTTCACACTCGAAG-3'
1		N1_XbaI rev	5'-CTATCTAGATTAGTCGATCACGAGGGTGC-3'
2	FRB_N2	KpnI_SP_myc_FRB fwd	5'-GCTTGGTACCATGAAGACGATCATCGCCCTGAGCTACATCTTCTGCCTGGTGTTCGCCGAGCAGAAGCTGATCTCCGAAGAGGACCTGGATCCACGAGTAGCCATCCTCTGGCATG – 3'
2		N2_FRB rev	5'-GATCATGTTCGGCGTAACCCCCGCCCCCGCCGCCCCCGCCTCGAGCTGCAGAATTCGAAGCTTCT-3'
2		N2_FRB fwd	5'-AGAAGCTTCGAATTCTGCAGCTCGAGGCGGGGGCGGCGGGGGCGGGGGTTACGCCGAACATGATC-3'
2		N2_XbaI rev	5'-GCCCTCTAGATTACGCCAGAATGCGTTCGC-3'
3	Mem_Link_N1 (MeN)	XhoI_N1 fwd	5'-GCGCTCGAGATGGTGTTCACCCTGGAAG-3'
3		N1_XbaI rev	5'-CTATCTAGATTAGTCGATCACGAGGGTGC-3'
4	Mem_Link_N2	XhoI_N2 fwd	5'-GCGCTCGAGGGCGTGACCCCTAACATGATC-3'
4		N2_XbaI rev	5'-CTATCTAGATCAGGCCAGAATTCTCTCGC-3'
5	N1_Link_Arr3	HindIII_N1 fwd	5'-GCGCTCGAGATGGTGTTCACCCTGGAAG-3'
5		N1_link_BglII rev	5'-CGAGATCTGAGTCCGTCGATCACGAGGGTGCCG-3'
6/7	N2_Link_Arr2/3 (ArC)	XhoI_N2 fwd	5'-AAGCTCGAGATGGGCGTGACCCCTAACATG-3'
6/7		N2_link_BglII rev	5'-CGAGATCTGAGTCCGGCCAGAATTCTCTCGCAC-3'
7	N2_Link_Arr2	Link_Arrestin2 fwd	5'-GGACTCAGATCTCGGCGAGCTCTCGACTCCATGGGCGACAAAGGGACCCGAG-3'
7		Arrestin2_NotI rev	5'- GATGCGGCCGCCTATCTGTTGTTGAGCTGTG-3'
8	ArC_P2A_MeN	NheI_N2 fwd	5'- GTAGCTAGCATGGGCGTGACCCCTAACATGATC -3'
8		Arr3_P2A_rev	5'- GAAGTTCGTGGCTCCGGATCCGCAGAGTTGATCATCATAGTC -3'
8		Arr3_P2A_fwd	5'-GACTATGATGATCAACTCTGCGGATCCGGAGCCACGAACTTC-3'
8		P2A fwd	5'- CGGAGCCACGAACTTCTCTCTGTTAAAGCAAGCAGGAG -3'
8		P2A rev	5'- CGGGGTTTTCTTCCACGTCTCCTGCTTGCTTTAACAGAGAG -3'
8		P2A mem fwd	5'- GTGGAAGAAAACCCCGGTCCTATGTGCTGTCTGAGAAGAAC -3'
8		P2A mem rev	5'-GTTCTTCTCAGACAGCACATAGGACCGGGGTTTTCTTCCAC-3'
8		N1 NotI rev	5'- GAGCGGCCGCTTAGTCGATCACGAGGGTGCCG -3'
9	N1_link_FYVE	3.1 KpnI N1 fwd	5'- TTTAAACTTAAGCTTGGTACATGGTGTTCACCCTGGAAG -3'
9		N1_link rev	5'- GAAGCTTGAGCTCGAGATCGTCGATCACGAGGGTGCCG -3'

9		Link_FYVE fwd	5’- GATCTCGAGCTCAAGCTTCAGAAACAGCCTACTTGGGTTC -3’
9		FYVE EcoRI 3.1 rev	5’- ACTGTGCTGGATATCTGCAGCTATTTACTAATAGTTTC -3’
10	**N2_link_FYVE**	3.1 KpnI N2 fwd	5’- TTTAAACTTAAGCTTGGTACATGGGCGTGACCCCTAAC -3’
10		N2_link rev	5’- GAAGCTTGAGCTCGAGATCGTCGATCACGAGGGTGCCG -3’
10	**D2R_link_N1**	Link_N1 fwd	5’- TTTAAACTTAAGCTTGGTACATGGGCGTGACCCCTAAC -3’
10		N1 XbaI rev	5’- GAAGCTTGAGCTCGAGATCGTCGATCACGAGGGTGCCG -3’

**Table 2**				
**PCR mix**	**50ul/tube**	**Cycles**	**Process**	**Temp C°**
10mM dNTP	1 µl	1	Denaturation	98
GC enhancer	10 µl	30	30 cycles (30sec/Kb)	98
GC buffer	10 µl			60
Q5 polymerase	0.5 µl			72
Primer fwd (0.1 µM)	2.5 µl	1	Final elongation	72
Primer rev (0.1 µM)	2.5 µl	1	Final hold	4
Insert (100 ng/ul)	1 µl			
H2O	22.5 µl			

**Table 3**
Construct 1 **FKBP_N1**
ATGAAGACGATCATCGCCCTGAGCTACATCTTCTGCCTGGTGTTCGCC<br>GAGCAGAAGCTGATCTCCGAAGAGGACCTGGATCCAGTGCAGGTGGAGACCATCTCTTCTGGAGACGGGCGCACCTTCCC<br>GAAGCGCGGCCAGACCTGCGTGGTACACTACACGGGGATGCTTGAAGATGGGAAGAAATTTGACTCCTCTCGGGACAGAA<br>ACAAGCCTTTTAAGTTTACACTAGGCAAGCAGGAGGTGATCCGAGGCTGGGAAGAAGGGGTAGCCCAGATGAGTGTGGG<br>CCAGAGAGCCAAACTGATAATCTCCCCAGACTATGCCTATGGAGCCACCGGGCACCCAGGCATCATCCCACCACATGCTACT<br>CTTGTTTTTGATGTGGAGCTTCTAAAACTGGAAAAGCTCGAGGCGGGGGCGGCGGGGGCGATGGTCTTCACACTCGAAGA<br>TTTCGTTGGGGACTGGCGACAGACAGCCGGCTACAACCTGGACCAAGTCCTTGAACAGGGAGGTGTGTCCAGTTTGTTTCA<br>GAATCTCGGGGTGTCCGTAACTCCGATCCAAAGGATTGTCCTGAGCGGTGAAAATGGGCTGAAGATCGACATCCATGTCAT<br>CATCCCGTATGAAGGTCTGAGCGGCGACCAAATGGGCCAGATCGAAAAAATTTTTAAGGTGGTGTACCCTGTGGATGATCA<br>TCACTTTAAGGTGATCCTGCACTATGGCACACTGGTAATCGACTAA
Construct 2 **FRB_N2**
ATGAAGACGATCATCGCCCTGAGCTACATCTTCTGCCTGGTGTTCGCCGAGCAGAAGCTGATCTCCGAAGAGGACCTGGAT<br>CCACGAGTAGCCATCCTCTGGCATGAGATGTGGCATGAAGGCCTAGAAGAGGCCTCTCGCTTGTACTTTGGGGAGAGGAA<br>CGTCAAAGGCATGTTTGAGGTGCTGGAGCCCCTGCATGCTATGATGGAACGCGGTCCCCAGACCCTGAAGGAAACGTCCTT<br>TAATCAGGCATATGGTCGAGATTTAATGGAGGCACAAGAATGGTGCCGAAAGTACATGAAATCAGGGAACGTCAAGGACC<br>TCACCCAAGCCTGGGACCTCTACTATCACGTGTTCAGACGGATCTCCAAGCAGAAGCTTCGAATTCTGCAGCTCGAGGCGG<br>GGGCGGCGGGGGCGGGGGTTACGCCGAACATGATCGACTATTTCGGACGGCCGTATGAAGGCATCGCCGTGTTCGACGG<br>CAAAAAGATCACTGTAACAGGGACCCTGTGGAACGGCAACAAAATTATCGACGAGCGCCTGATCAACCCCGACGGCTCCCT<br>GCTGTTCCGAGTAACCATCAACGGAGTGACCGGCTGGCGGCTGTGCGAACGCATTCTGGCGTAA
Construct 3 **Mem_Link_N1** (MeN)
ATGCTGTGCTGTCTGAGAAGAACCAAACAGGTTGAAAAGAATGATGAGGACCAAAAGATCATGGTGAGCAAGGGCGGCG<br>GAGGTTCCGGTGGGGGTGGCTCTGGCGGAGGTTCCGGTGGAGAGCTCCGCGGTGGAGAGCTCGAGATGGTGTTCACCCT<br>GGAAGATTTCGTGGGCGACTGGCGGCAGACCGCCGGCTACAATCTGGACCAGGTGCTGGAACAGGGCGGCGTGTCCAGC<br>CTGTTTCAGAACCTGGGCGTGTCCGTGACCCCCATCCAGAGAATCGTGCTGAGCGGCGAGAACGGCCTGAAGATCGACATC<br>CACGTGATCATCCCTTACGAGGGCCTGTCCGGCGACCAGATGGGCCAGATCGAGAAGATCTTTAAGGTGGTGTACCCCGTG<br>GACGACCACCACTTCAAAGTGATCCTGCACTACGGCACCCTCGTGATCGACTAA
Construct 4 **Mem_Link_N2**

ATGTGCTGTCTGAGAAGAACCAAACAGGTTGAAAAGAATGATGAGGACCAAAAGATCATGGTGAGCAAGGGCGGCGGAG<br>GTTCCGGTGGGGGTGGCTCTGGCGGAGGTTCCGGTGGAGAGCTCCGCGGTGGAGAGCTCGAGGGCGTGACCCCTAACAT<br>GATCGACTACTTCGGCAGACCCTACGAGGGAATCGCCGTGTTCGACGGCAAGAAAATCACCGTGACCGGCACCCTGTGGA<br>ACGGCAACAAGATCATCGACGAGCGGCTGATCAACCCCGACGGCAGCCTGCTGTTCAGAGTGACCATCAATGGCGTGACA<br>GGCTGGCGGCTGTGCGAGAGAATTCTGGCCTAG
Construct 5 **N1_Link_Arr3**
ATGGTGTTCACCCTGGAAGATTTCGTGGGCGACTGGCGGCAGACCGCCGGCTACAATCTGGACCAGGTGCTGGAACAGGG<br>CGGCGTGTCCAGCCTGTTTCAGAACCTGGGCGTGTCCGTGACCCCCATCCAGAGAATCGTGCTGAGCGGCGAGAACGGCC<br>TGAAGATCGACATCCACGTGATCATCCCTTACGAGGGCCTGTCCGGCGACCAGATGGGCCAGATCGAGAAGATCTTTAAGG<br>TGGTGTACCCCGTGGACGACCACCACTTCAAAGTGATCCTGCACTACGGCACCCTCGTGATCGACGGACTCAGATCTCGGC<br>GAGCTCTCGACTCCATGGGGGAGAAACCCGGGACCAGGGTCTTCAAGAAATCGAGTCCTAACTGCAAGCTCACCGTGTACT<br>TGGGCAAGCGGGACTTCGTAGATCACCTGGACAAAGTGGACCCTGTAGATGGCGTGGTGCTTGTGGACCCTGACTACCTG<br>AAGGACCGCAAAGTGTTTGTGACCCTCACCTGCGCCTTCCGCTATGGCCGTGAAGACCTGGATGTGCTGGGCTTGTCCTTCC<br>GCAAAGACCTGTTCATCGCCACCTACCAGGCCTTCCCCCCGGTGCCCAACCCACCCCGGCCCCCCACCCGCCTGCAGGACCG<br>GCTGCTGAGGAAGCTGGGCCAGCATGCCCACCCCTTCTTCTTCACCATACCCCAGAATCTTCCATGCTCCGTCACACTGCAG<br>CCAGGCCCAGAGGATACAGGAAAGGCCTGCGGCGTAGACTTTGAGATTCGAGCCTTCTGTGCTAAATCACTAGAAGAGAA<br>AAGCCACAAAAGGAACTCTGTGCGGCTGGTGATCCGAAAGGTGCAGTTCGCCCCGGAGAAACCCGGCCCCCAGCCTTCAG<br>CCGAAACCACACGCCACTTCCTCATGTCTGACCGGTCCCTGCACCTCGAGGCTTCCCTGGACAAGGAGCTGTACTACCATGG<br>GGAGCCCCTCAATGTAAATGTCCACGTCACCAACAACTCCACCAAGACCGTCAAGAAGATCAAAGTCTCTGTGAGACAGTA<br>CGCCGACATCTGCCTCTTCAGCACCGCCCAGTACAAGTGTCCTGTGGCTCAACTCGAACAAGATGACCAGGTATCTCCCAGC<br>TCCACATTCTGTAAGGTGTACACCATAACCCCACTGCTCAGTGACAACCGGGAGAAGCGGGGTCTCGCCCTGGATGGGAAA<br>CTCAAGCACGAGGACACCAACCTGGCTTCCAGCACCATCGTGAAGGAGGGTGCCAACAAGGAGGTGCTGGGAATCCTGGT<br>GTCCTACAGGGTCAAGGTGAAGCTGGTGGTGTCTCGAGGCGGGGATGTCTCTGTGGAGCTGCCTTTTGTTCTTATGCACCC<br>CAAGCCCCACGACCACATCCCCCTCCCCAGACCCCAGTCAGCCGCTCCGGAGACAGATGTCCCTGTGGACACCAACCTCATT<br>GAATTTGATACCAACTATGCCACAGATGATGACATTGTGTTTGAGGACTTTGCCCGGCTTCGGCTGAAGGGGATGAAGGAT<br>GACGACTATGATGATCAACTCTGCTAG
Construct 6 **N2_Link_Arr3**
ATGGGCGTGACCCCTAACATGATCGACTACTTCGGCAGACCCTACGAGGGAATCGCCGTGTTCGACGGCAAGAAAATCACC<br>GTGACCGGCACCCTGTGGAACGGCAACAAGATCATCGACGAGCGGCTGATCAACCCCGACGGCAGCCTGCTGTTCAGAGT<br>GACCATCAATGGCGTGACAGGCTGGCGGCTGTGCGAGAGAATTCTGGCCGGACTCAGATCTCGGCGAGCTCTCGACTCCA<br>TGGGGGAGAAACCCGGGACCAGGGTCTTCAAGAAATCGAGTCCTAACTGCAAGCTCACCGTGTACTTGGGCAAGCGGGAC<br>TTCGTAGATCACCTGGACAAAGTGGACCCTGTAGATGGCGTGGTGCTTGTGGACCCTGACTACCTGAAGGACCGCAAAGTG<br>TTTGTGACCCTCACCTGCGCCTTCCGCTATGGCCGTGAAGACCTGGATGTGCTGGGCTTGTCCTTCCGCAAAGACCTGTTCA<br>TCGCCACCTACCAGGCCTTCCCCCCGGTGCCCAACCCACCCCGGCCCCCCACCCGCCTGCAGGACCGGCTGCTGAGGAAGC<br>TGGGCCAGCATGCCCACCCCTTCTTCTTCACCATACCCCAGAATCTTCCATGCTCCGTCACACTGCAGCCAGGCCCAGAGGA<br>TACAGGAAAGGCCTGCGGCGTAGACTTTGAGATTCGAGCCTTCTGTGCTAAATCACTAGAAGAGAAAAGCCACAAAAGGA<br>ACTCTGTGCGGCTGGTGATCCGAAAGGTGCAGTTCGCCCCGGAGAAACCCGGCCCCCAGCCTTCAGCCGAAACCACACGCC<br>ACTTCCTCATGTCTGACCGGTCCCTGCACCTCGAGGCTTCCCTGGACAAGGAGCTGTACTACCATGGGGAGCCCCTCAATGT<br>AAATGTCCACGTCACCAACAACTCCACCAAGACCGTCAAGAAGATCAAAGTCTCTGTGAGACAGTACGCCGACATCTGCCT<br>CTTCAGCACCGCCCAGTACAAGTGTCCTGTGGCTCAACTCGAACAAGATGACCAGGTATCTCCCAGCTCCACATTCTGTAAG<br>GTGTACACCATAACCCCACTGCTCAGTGACAACCGGGAGAAGCGGGGTCTCGCCCTGGATGGGAAACTCAAGCACGAGGA<br>CACCAACCTGGCTTCCAGCACCATCGTGAAGGAGGGTGCCAACAAGGAGGTGCTGGGAATCCTGGTGTCCTACAGGGTCA<br>AGGTGAAGCTGGTGGTGTCTCGAGGCGGGGATGTCTCTGTGGAGCTGCCTTTTGTTCTTATGCACCCCAAGCCCCACGACC<br>ACATCCCCCTCCCCAGACCCCAGTCAGCCGCTCCGGAGACAGATGTCCCTGTGGACACCAACCTCATTGAATTTGATACCAA<br>CTATGCCACAGATGATGACATTGTGTTTGAGGACTTTGCCCGGCTTCGGCTGAAGGGGATGAAGGATGACGACTATGATG<br>ATCAACTCTGCTAG
Construct 7 **N2_Link_Arr2**
ATGGGCGTGACCCCTAACATGATCGACTACTTCGGCAGACCCTACGAGGGAATCGCCGTGTTCGACGGCAAGAAAATCACC<br>GTGACCGGCACCCTGTGGAACGGCAACAAGATCATCGACGAGCGGCTGATCAACCCCGACGGCAGCCTGCTGTTCAGAGT<br>GACCATCAATGGCGTGACAGGCTGGCGGCTGTGCGAGAGAATTCTGGCCGGACTCAGATCTCGGCGAGCTCTCGACTCCA<br>TGGGCGACAAAGGGACCCGAGTGTTCAAGAAGGCCAGTCCAAATGGAAAGCTCACCGTCTACCTGGGAAAGCGGGACTTT<br>GTGGACCACATCGACCTCGTGGACCCTGTGGATGGTGTGGTCCTGGTGGATCCTGAGTATCTCAAAGAGCGGAGAGTCTAT

GTGACGCTGACCTGCGCCTTCCGCTATGGCCGGGAGGACCTGGATGTCCTGGGCCTGACCTTTCGCAAGGACCTGTTTGTG
GCCAACGTACAGTCGTTCCCACCGGCCCCCGAGGACAAGAAGCCCCTGACGCGGCTGCAGGAACGCCTCATCAAGAAGCT
GGGCGAGCACGCTTACCCTTTCACCTTTGAGATCCCTCCAAACCTTCCATGTTCTGTGACACTGCAGCCGGGGCCCGAAGAC
ACGGGGAAGGCTTGCGGTGTGGACTATGAAGTCAAAGCCTTCTGCGCGGAGAATTTGGAGGAGAAGATCCACAAGCGGA
ATTCTGTGCGTCTGGTCATCCGGAAGGTTCAGTATGCCCCAGAGAGGCCTGGCCCCCAGCCCACAGCCGAGACCACCAGGC
AGTTCCTCATGTCGGACAAGCCCTTGCACCTAGAAGCCTCTCTGGATAAGGAGATCTATTACCATGGAGAACCCATCAGCGT
CAACGTCCACGTCACCAACAACACCAACAAGACGGTGAAGAAGATCAAGATCTCAGTGCGCCAGTATGCAGACATCTGCCT
TTTCAACACAGCTCAGTACAAGTGCCCTGTTGCCATGGAAGAGGCTGATGACACTGTGGCACCCAGCTCGACGTTCTGCAA
GGTCTACACACTGACCCCCTTCCTAGCCAATAACCGAGAGAAGCGGGGCCTCGCCTTGGACGGGAAGCTCAAGCACGAAG
ACACGAACTTGGCCTCTAGCACCCTGTTGAGGGAAGGTGCCAACCGTGAGATCCTGGGGATCATTGTTTCCTACAAAGTGA
AAGTGAAGCTGGTGGTGTCTCGGGGCGGCCTGTTGGGAGATCTTGCATCCAGCGACGTGGCCGTGGAACTGCCCTTCACC
CTAATGCACCCCAAGCCCAAAGAGGAACCCCCGCATCGGGAAGTTCCAGAGAACGAGACGCCAGTAGATACCAATCTCATA
GAACTTGACACAAATGATGACGACATTGTATTTGAGGACTTTGCTCGCCAGAGACTGAAAGGCATGAAGGATGACAAGGA
GGAAGAGGAGGATGGTACCGGCTCTCCACAGCTCAACAACAGATAG

Construct 8 N2_Link_Arr3_P2A_ mem _Link_N1

ATGGGCGTGACCCCTAACATGATCGACTACTTCGGCAGACCCTACGAGGGAATCGCCGTGTTCGACGGCAAGA
AAATCACCGTGACCGGCACCCTGTGGAACGGCAACAAGATCATCGACGAGCGGCTGATCAACCCCGACGGCAG
CCTGCTGTTCAGAGTGACCATCAATGGCGTGACAGGCTGGCGGCTGTGCGAGAGAATTCTGGCCGGACTCAGA
TCTCGGCGAGCTCTCGACTCCATGGGGGAGAAACCCGGGACCAGGGTCTTCAAGAAATCGAGTCCTAACTGCA
AGCTCACCGTGTACTTGGGCAAGCGGGACTTCGTAGATCACCTGGACAAAGTGGACCCTGTAGATGGCGTGGT
GCTTGTGGACCCTGACTACCTGAAGGACCGCAAAGTGTTTGTGACCCTCACCTGCGCCTTCCGCTATGGCCGTG
AAGACCTGGATGTGCTGGGCTTGTCCTTCCGCAAAGACCTGTTCATCGCCACCTACCAGGCCTTCCCCCCGGTGC
CCAACCCACCCCGGCCCCCCACCCGCCTGCAGGACCGGCTGCTGAGGAAGCTGGGCCAGCATGCCCACCCCTTC
TTCTTCACCATACCCCAGAATCTTCCATGCTCCGTCACACTGCAGCCAGGCCCAGAGGATACAGGAAAGGCCTG
CGGCGTAGACTTTGAGATTCGAGCCTTCTGTGCTAAATCACTAGAAGAGAAAAGCCACAAAAGGAACTCTGTG
CGGCTGGTGATCCGAAAGGTGCAGTTCGCCCCGGAGAAACCCGGCCCCCAGCCTTCAGCCGAAACCACACGCC
ACTTCCTCATGTCTGACCGGTCCCTGCACCTCGAGGCTTCCCTGGACAAGGAGCTGTACTACCATGGGGAGCCC
CTCAATGTAAATGTCCACGTCACCAACAACTCCACCAAGACCGTCAAGAAGATCAAAGTCTCTGTGAGACAGTA
CGCCGACATCTGCCTCTTCAGCACCGCCCAGTACAAGTGTCCTGTGGCTCAACTCGAACAAGATGACCAGGTAT
CTCCCAGCTCCACATTCTGTAAGGTGTACACCATAACCCCACTGCTCAGTGACAACCGGGAGAAGCGGGGTCTC
GCCCTGGATGGGAAACTCAAGCACGAGGACACCAACCTGGCTTCCAGCACCATCGTGAAGGAGGGTGCCAACA
AGGAGGTGCTGGGAATCCTGGTGTCCTACAGGGTCAAGGTGAAGCTGGTGGTGTCTCGAGGCGGGGATGTCT
CTGTGGAGCTGCCTTTTGTTCTTATGCACCCCAAGCCCCACGACCACATCCCCCTCCCCAGACCCCAGTCAGCCG
CTCCGGAGACAGATGTCCCTGTGGACACCAACCTCATTGAATTTGATACCAACTATGCCACAGATGATGACATT
GTGTTTGAGGACTTTGCCCGGCTTCGGCTGAAGGGGATGAAGGATGACGACTATGATGATCAACTCTGCGGAT
CCGGAGCCACGAACTTCTCTCTGTTAAAGCAAGCAGGAGACGTGGAAGAAAACCCCGGTCCTATGTGCTGTCT
GAGAAGAACCAAACAGGTTGAAAAGAATGATGAGGACCAAAAGATCATGGTGAGCAAGGGCGGCGGAGGTT
CCGGTGGGGGTGGCTCTGGCGGAGGTTCCGGTGGAGAGCTCCGCGGTGGAGAGCTCGAGATGGTGTTCACCC
TGGAAGATTTCGTGGGCGACTGGCGGCAGACCGCCGGCTACAATCTGGACCAGGTGCTGGAACAGGGCGGCG
TGTCCAGCCTGTTTCAGAACCTGGGCGTGTCCGTGACCCCCATCCAGAGAATCGTGCTGAGCGGCGAGAACGG
CCTGAAGATCGACATCCACGTGATCATCCCTTACGAGGGCCTGTCCGGCGACCAGATGGGCCAGATCGAGAAG
ATCTTTAAGGTGGTGTACCCCGTGGACGACCACCACTTCAAAGTGATCCTGCACTACGGCACCCTCGTGATCGA
CTAA

Construct 9 **N1_link_FYVE**

ATGGTGTTCACCCTGGAAGATTTCGTGGGCGACTGGCGGCAGACCGCCGGCTACAATCTGGACCAGGTGCTGGAACAGGG
CGGCGTGTCCAGCCTGTTTCAGAACCTGGGCGTGTCCGTGACCCCCATCCAGAGAATCGTGCTGAGCGGCGAGAACGGCC
TGAAGATCGACATCCACGTGATCATCCCTTACGAGGGCCTGTCCGGCGACCAGATGGGCCAGATCGAGAAGATCTTTAAGG
TGGTGTACCCCGTGGACGACCACCACTTCAAAGTGATCCTGCACTACGGCACCCTCGTGATCGACGATCTCGAGCTCAAGCT
TCAGAAACAGCCTACTTGGGTTCCTGATTCAGAAGCTCCAAACTGTATGAACTGCCAAGTCAAATTTACTTTTACCAAACGG

CGACACCATTGCCGAGCATGTGGGAAAGTATTTTGTGGTGTCTGTTGTAATAGGAAGTGTAAACTGCAATATCTAGAAAAG<br>GAAGCAAGAGTATGTGTAGTCTGCTATGAAACTATTAGTAAATAG
Construct 10 **N2_link_FYVE**
ATGGGCGTGACCCCTAACATGATCGACTACTTCGGCAGACCCTACGAGGGAATCGCCGTGTTCGACGGCAAGAAAATCACC<br>GTGACCGGCACCCTGTGGAACGGCAACAAGATCATCGACGAGCGGCTGATCAACCCCGACGGCAGCCTGCTGTTCAGAGT<br>GACCATCAATGGCGTGACAGGCTGGCGGCTGTGCGAGAGAATTCTGGCCGATCTCGAGCTCAAGCTTCAGAAACAGCCTA<br>CTTGGGTTCCTGATTCAGAAGCTCCAAACTGTATGAACTGCCAAGTCAAATTTACTTTTACCAAACGGCGACACCATTGCCG<br>AGCATGTGGGAAAGTATTTTGTGGTGTCTGTTGTAATAGGAAGTGTAAACTGCAATATCTAGAAAAGGAAGCAAGAGTAT<br>GTGTAGTCTGCTATGAAACTATTAGTAAATAG
Construct 11 SP_M1_**D2R_link_N1**
ATGAAGACGATCATCGCCCTGAGCTACATCTTCTGCCTGGTGTTCGCCGACTACAAGGACGATGATGACGCCATGGATCCA<br>CTGAATCTGTCCTGGTATGATGATGATCTGGAGAGGCAGAACTGGAGCCGGCCCTTCAACGGGTCAGACGGGAAGGCGGA<br>CAGACCCCACTACAACTACTATGCCACACTGCTCACCCTGCTCATCGCTGTCATCGTCTTCGGCAACGTGCTGGTGTGCATG<br>GCTGTGTCCCGCGAGAAGGCGCTGCAGACCACCACCAACTACCTGATCGTCAGCCTCGCAGTGGCCGACCTCCTCGTCGCC<br>ACACTGGTCATGCCCTGGGTTGTCTACCTGGAGGTGGTAGGTGAGTGGAAATTCAGCAGGATTCACTGTGACATCTTCGTC<br>ACTCTGGACGTCATGATGTGCACGGCGAGCATCCTGAACTTGTGTGCCATCAGCATCGACAGGTACACAGCTGTGGCCATG<br>CCCATGCTGTACAATACGCGCTACAGCTCCAAGCGCCGGGTCACCGTCATGATCTCCATCGTCTGGGTCCTGTCCTTCACCA<br>TCTCCTGCCCACTCCTCTTCGGACTCAATAACGCAGACCAGAACGAGTGCATCATTGCCAACCCGGCCTTCGTGGTCTACTCC<br>TCCATCGTCTCCTTCTACGTGCCCTTCATTGTCACCCTGCTGGTCTACATCAAGATCTACATTGTCCTCCGCAGACGCCGCAA<br>GCGAGTCAACACCAAACGCAGCAGCCGAGCTTTCAGGGCCCACCTGAGGGCTCCACTAAAGGAGGCTGCCCGGCGAGCCC<br>AGGAGCTGGAGATGGAGATGCTCTCCAGCACCAGCCCACCCGAGAGGACCCGGTACAGCCCCATCCCACCCAGCCACCAC<br>CAGCTGACTCTCCCCGACCCGTCCCACCATGGTCTCCACAGCACTCCCGACAGCCCCGCCAAACCAGAGAAGAATGGGCAT<br>GCCAAAGACCACCCCAAGATTGCCAAGATCTTTGAGATCCAGACCATGCCCAATGGCAAAACGCGTACCTCCCTCAAGACC<br>ATGAGCCGTAGGAAGCTCTCCCAGCAGAAGGAGAAGAAAGCCACTCAGATGCTCGCCATTGTTCTCGGCGTGTTCATCATC<br>TGCTGGCTGCCCTTCTTCATCACACACATCCTGAACATACACTGTGACTGCAACATCCCGCCTGTCCTGTACAGCGCCTTCAC<br>GTGGCTGGGCTATGTCAACAGCGCCGTGAACCCCATCATCTACACCACCTTCAACATTGAGTTCCGCAAGGCCTTCCTGAAG<br>ATCCTCCACTGCGCTACCGGACTCAGATCTCGAGCTCAAGCTTCGAATTCTGCAGTCGACGGCACCGCGGGCCCGGTCGCC<br>ACCATGGTGTTCACCCTGGAAGATTTCGTGGGCGACTGGCGGCAGACCGCCGGCTACAATCTGGACCAGGTGCTGGAACA<br>GGGCGGCGTGTCCAGCCTGTTTCAGAACCTGGGCGTGTCCGTGACCCCCATCCAGAGAATCGTGCTGAGCGGCGAGAACG<br>GCCTGAAGATCGACATCCACGTGATCATCCCTTACGAGGGCCTGTCCGGCGACCAGATGGGCCAGATCGAGAAGATCTTTA<br>AGGTGGTGTACCCCGTGGACGACCACCACTTCAAAGTGATCCTGCACTACGGCACCCTCGTGATCGACTAA
