# Supplementary material for: A novel luminescence-based β-arrestin recruitment assay for unmodified receptors
Source: J Biol Chem. 2021 Mar 5;296:100503. doi: 10.1016/j.jbc.2021.100503 (PMC8027564; doi:10.1016/j.jbc.2021.100503)
Supplement: Figures S1 to S4 [file mmc2.pdf]

Supplementary Figures

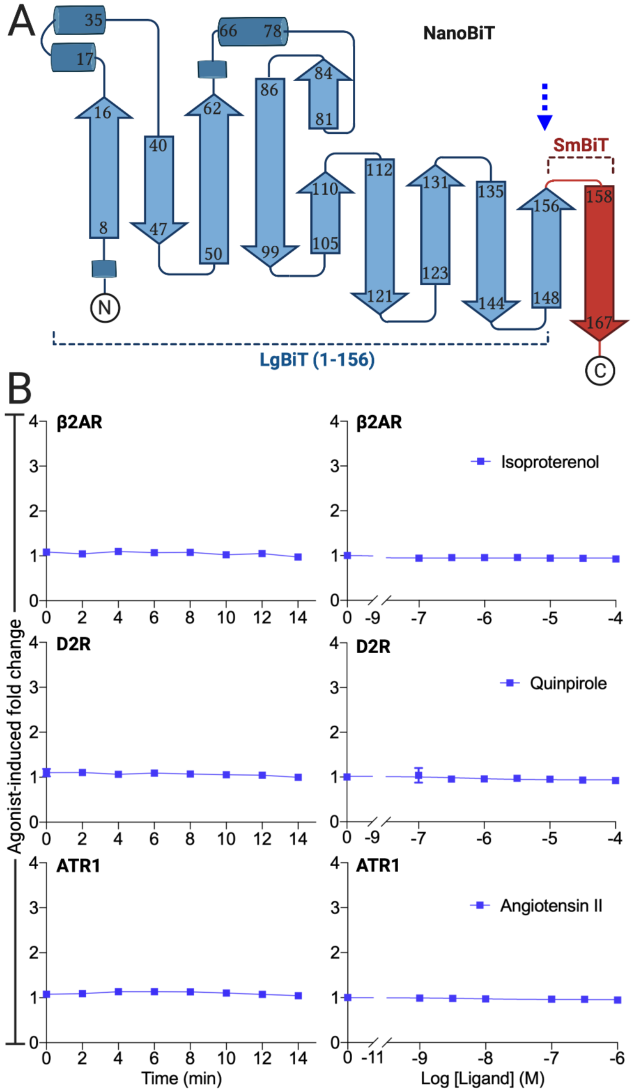

Supplementary Figure 1 - β -arrestin membrane recruitment assay tested with NanoBiT
A) NanoLuc topology showing the LgBiT and SmBiT fragments that constitute the NanoBit system in blue and red, respectively. The topology diagram was derived from that generated by Pro-origami (34). B) NanoBiT tested as an β-arrestin 2 membrane-recruitment assay where the SmBiT was tethered to the membrane by fusing it to the doubly palmitolyated fragment of GAP43 and the LgBiT was fused to the N terminus of β-arrestin 2. Three different receptors were tested: Angiotensin II Type 1 receptor (AT1R), beta 2-adrenergic receptor (β2AR) and the dopamine D2 receptor (D2R). Shown are time course curves after agonist addition (100 μM isoproterenol and dopamine and 1 μM Ang II was used) on the left and dose response curves after 20 min on the right, with the agonist isoproterenol, dopamine and angiotensin II used for β2AR, D2R and AT1R respectively.

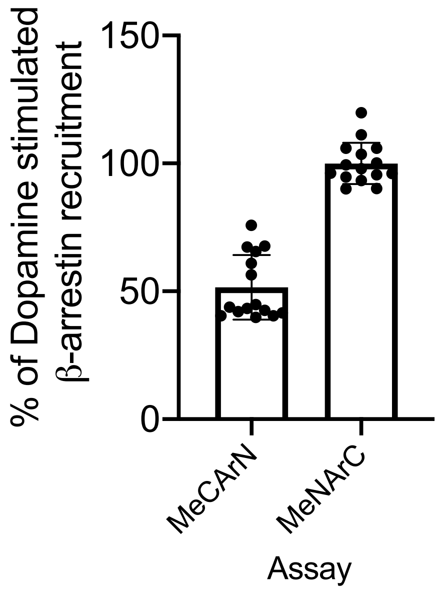

Supplementary figure 2. Comparison of the NanoLuc split orientation with membrane β-arrestin recruitment in Hek293 cells transiently cotransfected with D2R and GRK2. MeCArN: C-terminal NanoLuc split tethered to the membrane and N-terminal NanoLuc split tethered to β-arrestin2 MeNArC: N-terminal NanoLuc split tethered to the membrane and C-terminal NanoLuc split tethered to β-arrestin2. Data is shown as % of 10 μM dopamine stimulated β-arrestin recruitment in the MeNArC orientation; mean±SD MeCArN: 51.53±12.63 and MeNArC: 100±8.12. Data is compiled of 5 individual experiments performed in triplicate.

A

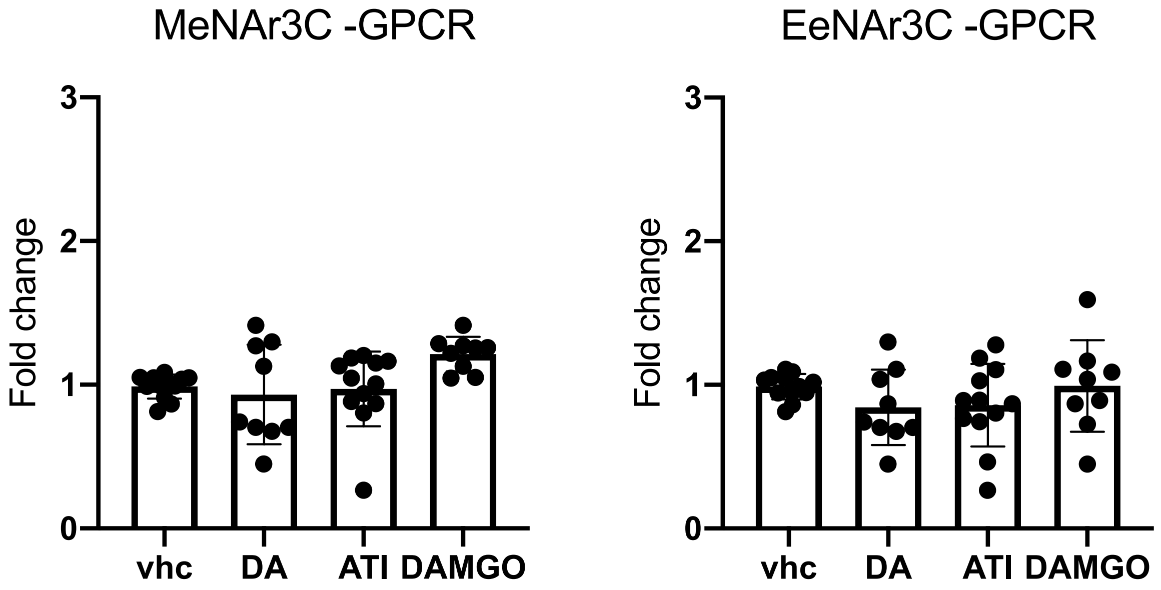

Supplementary figure 3. Negative controls of the MeNArC and EeNArC assay transiently transfected into Hek293 cells without cotransfection of the cognate GPCRs. Tested with agonists for D2R (10 μM dopamine), AT1R (10 μM Ang II) and MOR (10 μM DAMGO).

ATGGTGTTCACCCTGGAAGATTTCGTGGGCGACTGGCGGCAGACCGCCGGCTACAAT
CTGGACCAGGTGCTGGAACAGGGCGGCGTGTCCAGCCTGTTTCAGAACCTGGGCGT
GTCCGTGACCCCCATCCAGAGAATCGTGCTGAGCGGCGAGAACGGCCTGAAGATCG
ACATCCACGTGATCATCCCTTACGAGGGCCTGTCCGGCGACCAGATGGGCCAGATC
GAGAAGATCTTTAAGGTGGTGTACCCCGTGGACGACCACCACTTCAAAGTGATCCTG
CACTACGGCACCCTCGTGATCGACGGCGTGACCCCTAACATGATCGACTACTTCGGC
AGACCCTACGAGGGAATCGCCGTGTTCGACGGCAAGAAAATCACCGTGACCGGCAC
CCTGTGGAACGGCAACAAGATCATCGACGAGCGGCTGATCAACCCCGACGGCAGCC
TGCTGTTCAGAGTGACCATCAATGGCGTGACAGGCTGGCGGCTGTGCGAGAGAATT
CTGGCCTGA

Supplementary Figure 4 – codon optimized NanoLuc

NanoLuc codon optimized for human expression using Geneart, changing 87 bp in total.
